# Functional Coatings for Orthodontic Archwires—A Review

**DOI:** 10.3390/ma13153257

**Published:** 2020-07-22

**Authors:** Justyna Bącela, Magdalena Beata Łabowska, Jerzy Detyna, Anna Zięty, Izabela Michalak

**Affiliations:** 1 Department of Mechanics, Materials and Biomedical Engineering, Faculty of Mechanical Engineering, Wrocław University of Science and Technology, Smoluchowskiego 25, 50-372 Wrocław, Poland; justyna.bacela@pwr.edu.pl (J.B.); jerzy.detyna@pwr.edu.pl (J.D.); anna.ziety@pwr.edu.pl (A.Z.); 2 Department of Advanced Material Technologies, Faculty of Chemistry, Wrocław University of Science and Technology, Smoluchowskiego 25, 50-372 Wrocław, Poland; izabela.michalak@pwr.edu.pl

**Keywords:** archwire, coating, antiadherent, antimicrobial, surface modified, orthodontics

## Abstract

In this literature review, the current state-of-art of coatings for orthodontic archwires’ increasing antimicrobial and relevant mechanical properties, such as surface topography, friction or corrosion resistance, has been presented. There is a growing request for orthodontic appliances, therefore, most researchers focus on innovative functional coatings to cover orthodontic archwires and brackets. Orthodontic appliances are exposed to the unfavorable oral cavity environment, consisting of saliva flow, food, temperature and appliance force. As a consequence, friction or biocorrosion processes may occur. This can affect the functionality of the orthodontic elements, causing changes in their microstructure, surface topography and mechanical properties. Furthermore, the material which the orthodontic archwire is made from is of particular importance in terms of the possible corrosion resistance. This is especially important for patients who are hypersensitive to metals, for example, nickel, which causes allergic reactions. In the literature, there are some studies, carried out in vitro and in vivo, mostly examining the antibacterial, antiadherent, mechanical and roughness properties of functional coatings. They are clinically acceptable but still some properties have to be studied and be developed for better results. In this paper the influence of additives such as nanoparticles of silver and nitrogen-doped TiO_2_ applied on orthodontic brackets by different methods on the antimicrobial properties was analyzed. Future improvement of coating techniques as well as modification of the archwire composition can reduce the release of nickel ions and eliminate friction and bacterial adhesion problems, thus accelerating treatment time.

## 1. Introduction

Displacement of the teeth might have major impact on good oral and dental health, as well as on a person’s appearance, contributing to an attractive smile, which can improve psychological self-esteem and self-confidence [1]. Orthodontic treatment is one of the most efficient paths to revise the position of the teeth [2]. The demand for orthodontic appliances is growing in various groups of age [3].

The mechanical basis of orthodontic treatment consists of the assumption that accumulated elastic energy can be transformed into mechanical work by the movement of teeth. Therefore, the perfect tooth movement control requires the application of a system of specific forces appropriately supported by accessories such as orthodontic archwires [4,5]. In the oral cavity environment, orthodontic archwires are affected by many factors. These include saliva flow and ingested fluids and food, temperature fluctuations, and masticatory and appliance force. As a consequence, friction or biocorrosion processes may occur. These difficult conditions affect the functionality of the orthodontic elements, causing changes in their microstructure, surface topography and mechanical properties [6,7].

Dental plaque and a biofilm are regarded as the main contributing factors of periodontal diseases and dental caries. White spot lesions (WSL) or carious demineralization are prevalent unwanted side-effects of orthodontic treatment [8,9]. Based on the dental literature, orthodontic treatment with fixed appliances may increase plaque accumulation and a heightened amount of *Streptococcus mutans* (*S. mutans*; SM) bacteria and lactobacilli, which are recognized as the primary pathogens in dental caries. The use of an orthodontic appliance contributes to formation of new surfaces exposed to the plaque development, thus increasing the amount of microorganisms in the oral environment. Fixed orthodontic appliances components for instanece brackets, archwires and bands are used as plaque-retentive niches hindering effective oral hygiene and resulting in a high cariogenic challenge [10,11]. The prevention of these changes is an essential issue for orthodontists. The mechanical and medical characteristics of archwires are important due to their influence on probable allergic or corrosion issues during the treatment [12,13].

Corrosion behavior is often one of the key characteristic of metallic material, as cytotoxicity and biocompatibility are related to the corrosion process products. According to reports, 46% of patients who are treated with fixed appliances, will suffer from demineralization within 12 months [14,15]. Much research has been done on reducing the friction between brackets and archwires using surface treatments such as ion implantation, polyethylene coating and poly(tetrafluoroethylene) coating on the archwires and/or brackets. One of the surface-modified coatings is a diamond-like carbon (DLC) coating, which has been applied in many industrial applications. It confers excellent characteristics such as low friction coefficients, extreme surface hardness, high wear resistance, chemical inertness, as well as a good biocompatibility. Although these methods effectively decrease the ligature force and thereby the friction, control of orthodontic tooth movement is more demanding due to the reduced contact between the wire and the bracket [16]. Moreover, when rigid heavy wire is used at high angulation, the friction between archwire and bracket is comparable to that for conventional brackets. However, the durability of the coatings and the friction reduction are clinically insufficient [17].

Nickel–titanium (NiTi) alloy is the most effective shape memory alloy and has been currently used as a material for orthodontic archwires production. It is differentiated by an exceptional combination of properties, including shape memory effect and superelasticity, which makes it attractive for biomedical applications. NiTi orthodontic wires are used together with brackets to create orthodontic forces affecting tooth movement, especially during the first stage of the orthodontic treatment. Nonetheless, NiTi is unstable during a long-term use. Since the NiTi appliances are eroded by active saliva, the release of corroded material and Ni ions into oral cavities may result in considerable health problems, such as allergenic, toxic and carcinogenic reactions [18]. By comparing the surface topography of stainless steel and NiTi archwires, an increase in roughness was observed for the NiTi archwires that is in line with the research carried out by Yu et al. [19] and Sheibaninia et al. [20].

The main purpose of this paper is to the review coating techniques and materials used in these processes, as well as different surface treatments carried out on orthodontic materials to achieve antimicrobial and relevant mechanical properties. To select the appropriate protective coating, a thorough knowledge of biomechanical properties of selected material archwires is required. In the past, there were attempts to improve the mechanical properties of orthodontic wires using variable types of alloys and surface treatment of archwires. These tests were satisfactory to some extent [21]. Coating the orthodontic archwires is one such attempt that is still under research. In the present paper, the novel methods of coating orthodontic wires are presented.

Coating is one of the available ways to modify the material surface. The application of coatings to the surface of orthodontic archwires using different techniques and materials and through modifying their surface is one of the strategies devised to reinforce the mechanical, as well as biological, properties of metallic materials utilized in orthodontics. There are many types of coatings depending on their purpose. The coating of orthodontic archwires is undertaken in order to impact their surface characteristics, and, hence, the properties of archwires inter alia their surface roughness [22,23,24], thickness [25], mechanical and frictional properties [22,24,25,26,27,28,29,30,31], corrosiveness [27], bacterial adhesion [23,32] and coating stability [26,33]. Different coating techniques and materials have been used to enhance surface properties. Nevertheless, some problems with coatings have been observed, mainly wear of the coating and its delamination. Investigations are continuing to find appropriate materials and techniques with the objective of improving the properties of metallic biomaterials [34].

**Figure 1:**
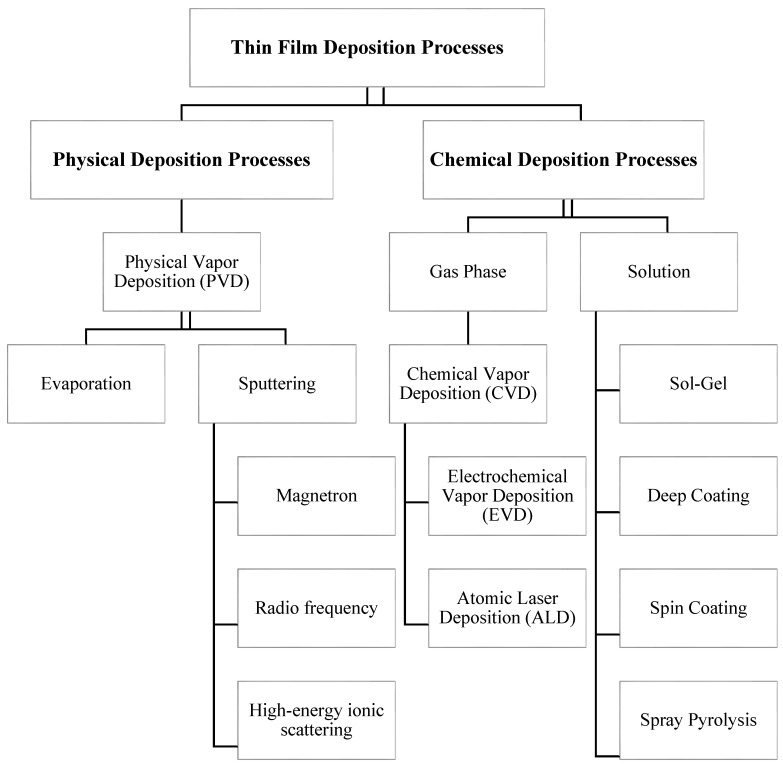
Classification of thin film coating techniques [34].

Figure 1 shows the coating methods classification used for thin film deposition. Several techniques have been applied in orthodontics to enhance the surface properties of these materials.

### 1.1. Physical Deposition Processes

#### 1.1.1. Physical Vapor Deposition (PVD)

**Figure 2:**
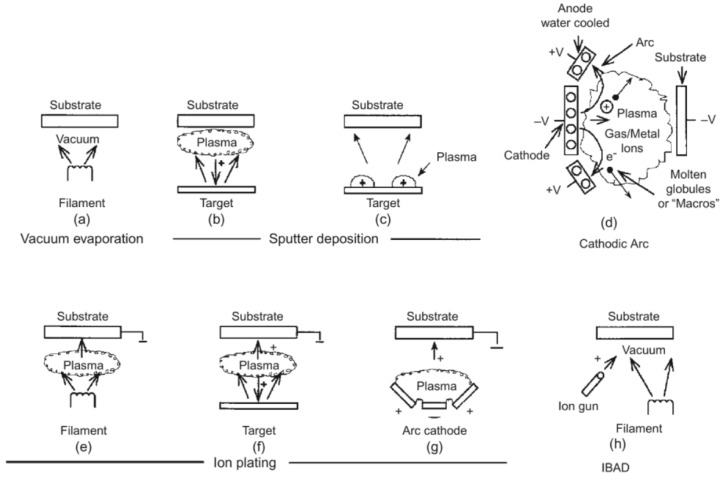
PVD Processing Techniques including categories of vacuum evaporation (**a**), sputter deposition in a plasma environment (**b**,**c**) and in a vacuum (**d**), ion planting in a plasma environment with a thermal evaporation source (**e**), with a sputtering source (**f**) and with an arc vaporization source (**g**) and ion-assisted deposition (IBAD) (**h**) [35].

The physical vapor deposition process is atomistic deposition process wherein material is evaporated from a liquid or solid source in the form of atoms or molecules and transported in the form of vapor through a vacuum or low-pressure gaseous (or plasma) environment to the substrate, where it liquefies. This process is suitable to deposit coatings with thicknesses ranging from several nanometers to thousands of nanometers for graded composition deposits, multilayer films, very thick deposits and freestanding structures. Typical PVD deposition rates are 1–10 nanometers per second [35]. Physical vapor deposition can be divided into several categories: evaporation (vacuum deposition), arc vapor deposition, sputter deposition and ion planting, as shown in Figure 2 [34,35]. The most common physical vapor deposition processes are sputtering and evaporation.

#### 1.1.2. Evaporation

Evaporation, which is sometimes called vacuum deposition, is the simplest method of physical vapor deposition. This technique is conducted in a vacuum system, where the material is heated to temperatures close to its melting or sublimation point [34,36]. Tripi et al. [37] utilized this technique for coating endodontic NiTi files. The application of this method to cover orthodontic metallic materials has not been described in the literature.

#### 1.1.3. Physical Sputtering

Sputter deposition is the deposition of particles vaporized from a surface by the physical sputtering process. In this process, atoms’ or molecules’ vapor from a solid surface by momentum transfers from bombarding energetic atomic-sized particles. These particles are ions of a gaseous material accelerated in an electric field [34,36,37]. Physical sputtering has a number of methods, for example, radiofrequency (RF) magnetron sputtering, as well as high-energy ionic scattering.

Radiofrequency magnetron sputtering is an effective technique on account of its inherent versatility, low-temperature deposition and uniform surface coverage [15,38,39]. The sputtering method removes surface atoms from a solid cathode by bombarding it with positive ions from an inert gas, for example argon, discharges, and next deposits them on the surface to create a thin layer. Substrates are positioned in a vacuum chamber and are pumped down to a specified process pressure. If a negative charge occurs on the target material, sputtering starts. This causes plasma or glow discharge. Positively charged ions of gas created in the plasma area are attracted extremely quickly to the negatively biased target plate. This interaction causes a momentum transfer and ejects atomically sized particles from the target. Those particles are accumulated on the substrate surface as a thin layer [40]. Shah et al. [40] used the radiofrequency magnetron sputtering to apply photocatalytic TiO_2_ to stainless steel orthodontic brackets in order to evaluate the antibacterial and antiadherent properties of these compounds against *Lactobacillus acidophilus*. The main advantage of this method is the ability to apply a thin, inorganic, nanoparticle coating with an adjustable shape and size by tuning preparation parameters [41].

### 1.2. Chemical Deposition Processes

#### 1.2.1. Chemical Vapor Deposition (CVD)

**Figure 3:**
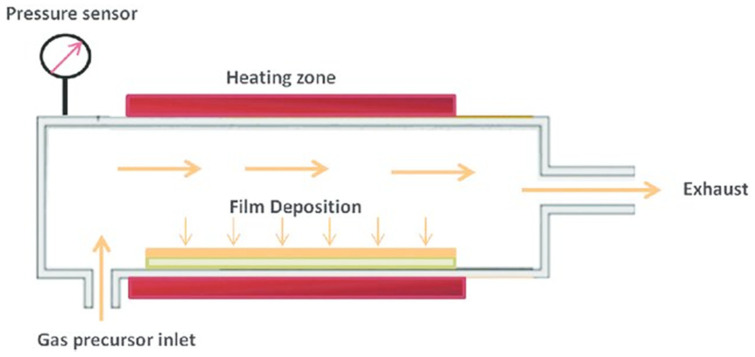
Schematic diagram of a chemical vapor deposition (CVD) system [44].

In chemical vapor deposition, a gas precursor flows to a chamber which contains one or more heated substrates to be covered. This process is accompanied by the manufacture of chemical by-products and their further excretion from the chamber together with unreacted precursor gases. Traditional methods of CVD require high temperatures, necessary for the desired reaction (600–900 °C) to take place, which significantly limit the scope of application of these methods. The schematic graph of a chemical vapor deposition is shown in Figure 3. There are several types of CVD technique, including low-pressure chemical vapor deposition, atmospheric pressure chemical vapor deposition, metal–organic chemical vapor deposition, laser chemical vapor deposition or photochemical vapor deposition. The main advantages of CVD are the coatings obtained by this process are conformal, which means that 6he thickness of the layer on the top is comparable to the film thickness on the sidewalls, presenting the high deposition rates and a wide range of materials can be used. Moreover, the objects can be deposited with a high purity level. However, the CVD has several disadvantages; the main ones are the properties of precursors, because they have to be volatile at room temperatures. Another one is the fact that precursors are toxic, dangerous and expensive, explosive and corrosive [34,42,43].

#### 1.2.2. Electrodeposition

The electrodeposition is a technique, in which the surface to be coated is manufactured using the negative electrode or cathode in a cell which includes an electrolyte. The electrolyte has to enable the flow of an electrical current. Usually, the electrolyte is a water solution of a deposited metal salt and it is kept at a controlled temperature. An anode completes the electrical circuit and is located at a short distance from the cathode. Positively charged ions in the electrolyte move in the cathode direction when it is powered by low and direct voltage current. Further, these ions are transformed to metal atoms and deposited on the cathode [45]. Redlich et al. [46] applied this technique to cover orthodontic archwires in order reduce friction. They used a Ni film impregnated with inorganic fullerene-like nanospheres of tungsten disulphide. The results indicated a considerable reduction in friction for coated versus uncoated arches. Zein El Abedin et al. [47] used electrodeposition to electroplate tantalum on NiTi alloys and carried out electrochemical tests in 3.5% NaCl solutions. The results show that coated samples were characterized by greater resistance to corrosion compared to uncoated alloys. Qiu et al. [48] applied hydroxyapatite and hydroxyapatite/zirconia composite coatings on NiTi alloys by electrodeposition. The aim of their study was to assess the corrosion resistance. The result showed that, in a simulated body fluid after the application of such a coating, the corrosion resistance was notably enhanced.

#### 1.2.3. Sol-Gel

The sol-gel process is a long-established industrial method for the creation of colloidal nanoparticles (NPs) from the liquid phase, which has been further developed for advanced nanomaterials and coatings’ production. It is a chemical method that allows the synthesis of glass/ceramic, glass and ceramic materials at significantly lower temperatures than CVD, PVD or plasma spray and diverse shapes may be obtained, such as nanospheres or monoliths [49,50].

**Figure 4:**
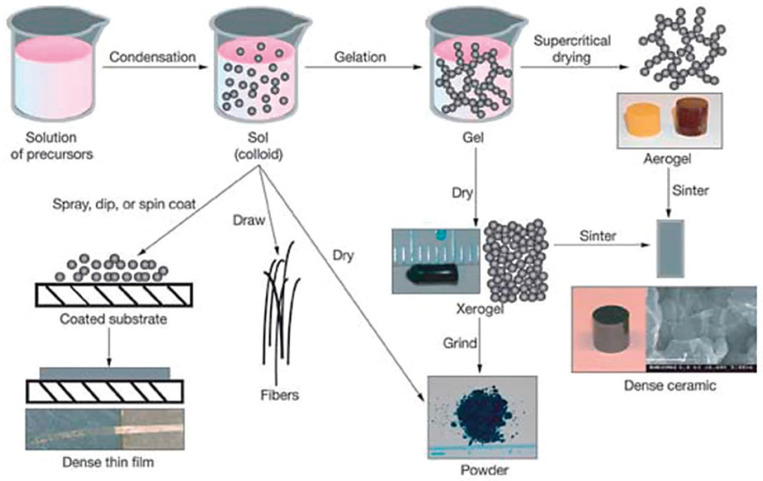
Stages of the sol-gel process [56].

The sol-gel processes are suitable for the synthesis of oxide nanoparticles and composite nanopowders. In general, the sol-gel method includes the transformation of a solution system from a liquid sol into a solid gel, and finally a dry gel is obtained. The initial materials used in the sol preparation are mostly inorganic metal salts or metal organic compounds such as metal alkoxides. The formation of sol is a stable dispersion of colloidal particles or polymers in solvent. Usually, the precursor is subjected to a series of hydrolysis and polymerization reactions to form a colloidal suspension or sol [51,52,53,54,55]. In the next step, the process allows the production of materials in different forms, such as monolith, fibers, film and monosized powders. The second stage after obtaining a homogeneous sol is gelation, i.e., a condensation process consisting of forming a bond network. A phase of gel is obtained by a three-dimensional continuous network, which includes a liquid phase, or by polymer chains bonding. The network, in a colloidal gel, consists of colloidal particle agglomerates. In general, van der Waals forces, as well as hydrogen bonds dominate the interactions between the sol’s particles. Ripening is the next stage of the sol-gel process. As a result, the three-dimensional structure of the gel is expanded, which is connected with the release of water and alcohol. The resulting gel is then subjected to a drying process, during which its final structure is formed. The final stage of the sol-gel process is firing, the aim of which is to remove the remaining hydroxyl or organic groups [34,56,57]. Figure 4 presents a route outline of this mechanism.

The film or coating depositions are representatives of the oldest commercial use of the sol-gel technique. Nowadays, sol-gel thin film coatings are extensively studied for different applications such as optical and protective coatings, high or low dielectric constant films, passivation and planarization layers, sensors, inorganic membranes, superconducting films, electro-optic and nonlinear optical layers, electrochromic, semiconducting anti-static coating, superconducting layer, strengthening films and ferroelectrics [34,56,58].

The main advantages of the sol-gel method are [54,59,60]:

- Better homogeneity of structure;
- High purity of starting materials;
- Sol-gels are obtained at low temperature, which results in trapping particles with low chemical durability;
- Possibility to control the porosity and structure of a fixed, three-dimensional network of gels;
- Possibility to control the conductivity of the resulting material.

This method was used by Chun et al. [11] to evaluate the antibacterial and antiadherent properties of stainless steel orthodontic archwires. They applied photocatalytic titanium oxide (TiO_2_) to coat the surface of wires. The results showed an antiadherent effect against some bacterial strains compared with the uncoated wires. This investigation also confirmed that photocatalytic TiO_2_ may be used to protect against dental plaque development during orthodontic treatment. In 2015, Syed et al. [21] developed a novel method of coating orthodontic archwires with a uniform and smooth nanoparticle film using nanocremics. The used method in this investigation was a sol-gel thin film dip coating.

## 2. Materials and Method

An advanced literature search was performed using Medline (PubMed) database from the period 2004–2020. The literature review was undertaken for reviewed papers published in English. The systematic search was conducted for the keywords “coated archwires”, “coated brackets”, “orthodontic coatings”. Articles recovered from the electronic search were manually searched for associated references. The full text of every articles detected by the electronic and manual searches was reviewed and evaluated as appropriate. The following inclusion and exclusion criteria were applied.

Inclusion criteria:

1. Both in-vivo and in-vitro studies were included;
2. Studies exclusively on functional coatings for orthodontic archwires and brackets;
3. Studies on different types of archwires and brackets;
4. Studies in English language only;
5. Any type of studies, including review articles, descriptive studies and case control trials, were considered.

Exclusion criteria: studies on lingual orthodontic wires.

The full text of 74 articles, after duplicate removal and applying the inclusion and exclusion criteria, were assessed.

## 3. Results

In order to perform a literature review, this article was divided into two sections, according to the characteristics and properties of each type of orthodontic functional coating and material. The first one contains the antiadherent properties and antibacterial effects of the orthodontic coatings covering archwires and brackets and orthodontic materials. The second one considers the mechanical properties of orthodontic elements used in the oral cavity.

### 3.1. Antimicrobial Properties of Coating for Orthodontic Elements

The appearance and adhesion of microorganisms on orthodontic appliances surfaces results in significant complications, such as enamel demineralization or White Spot Lesions around them. Therefore, applying antimicrobial coatings, which protect or obstruct the development of harmful microorganisms, has become popular in recent years. Several approaches have been applied to achieve this antibacterial behavior.

The first one is to create an antiadhesive surface, which protects against bacteria adherence and the consecutive biofilm formation using chemical or physical modifications. The second one is to create coatings with the possibility of releasing antibacterial compounds. This consequently reduces potential toxicity and resistance development. The third one is the use of biocidal coatings where substances with antibacterial activity are incorporated into the surface to ensure a permanent protection effect [61,62,63]. Different studies of the antimicrobial properties of orthodontic materials and coatings conducted in recent years are presented in Table 1.

#### 3.1.1. Common oral Microbes

The oral cavity has the most diverse environment from all areas throughout the body. The recent literature [75,76,77] indicates that, only in the mouth, there are over 700 types of microorganisms, of which only 250 are completely identified. Most bacterial species appear in the certain areas of an oral cavity that have conducive conditions for them. *Streptococcus mutans* most often initiates the development of White Spot Lesions, while increased colonization of *Porphyromonas gingivalis* bacteria may indicate periodontitis [75,76,77,78].

The most frequently isolated bacteria from the human oral cavity are the following strains: *Lactobacillus acidophilus, Porphyromonas gingivalis, Aggregatibacter actinomycetemco-mitans* and yeast *Candida albicans* [11,40,67,69,71,79,80]. Numerous investments have been carried out to create an antibacterial orthodontic adhesive system to protect the enamel demineralization at the junction. One of the methods is to involve different antimicrobial agents, such as chlorhexidine and fluoride, into orthodontic adhesives by physical stirring [81,82,83]. Nevertheless, these substances have some weaknesses, such as the short-term release of antimicrobial compounds and limited mechanical properties. Therefore, SM adhesion to orthodontic materials may be considered as a fundamental factor in the pathogenesis of enamel demineralization in the orthodontic treatment [23,84]. Among several pathogenic organisms which accumulate and colonize in the form of plaque, lactobacilli are not very important in initiation, but are significant during the lesion process progression. With an established low pH, the number of lactobacilli grows and the amount of *S. mutans* bacteria reduces; this results in demineralization of the teeth once White Spot Lesions are established. Preventing WSL is an relevant issue for orthodontists, because it is unhealthy, unaesthetic and potentially irreversible [40,80].

#### 3.1.2. Reduction in the Initial Adhesion of Microorganisms on Orthodontic Appliances

Nowadays, there is an urgent need for dental biomaterials with antiadhesion effects against bacteria. The reduction in bacterial adhesion will primarily be more effective than the application of bactericidal agents in dental appliances that inhibits the proliferation of accumulated bacteria [85,86,87]. In order to endow adhesives with antibacterial properties, several agents (e.g., chlorhexidine and fluorine-containing agent) were mixed with adhesives [87]. However, new antimicrobial agents are still in demand. For this reason, Peng et al. [86] proposed using an antiadhesive orthodontic appliance, which is grafted with a long-chain polyethylene glycol (PEG) coating. Due to the enhanced hydrophilicity of modified stainless steel archwires, an effective reduction in *Streptococcus mutans* adhesion was achieved for a long period of time. Altmann et al. [85] developed an orthodontic adhesive containing 1,3,5-triacryloylhexahydro-1,3,5-triazine with antibacterial activity (*S. mutans*). Degrazia et al. [88] used the orthodontic adhesive containing triazine and niobium phosphate bioglass around brackets. This approach resulted in the inhibition of *S. mutans* and total *Streptococci* increase and, additionally, an anti-demineralization effect. Yu et al. [87] reported that resin-based orthodontic adhesives that have been used for the bonding of the brackets to the enamel, due to their rough surface, can facilitate the adhesion of microorganisms. Therefore, it was proposed to add to this adhesive the antibacterial monomer 2-methacryloxylethyl hexadecyl methyl ammonium bromide, which exhibited a significant inhibition of *S. mutans* growth. Imazato, et al. [89] immobilized, in a resin-based material—monomer methacryloyloxydodecylpyridinium, bromide which exhibited antibacterial activity against *Streptococcus mutans*, *Actinomyces viscosus* and *Lactobacillus casei*. Novel polymethyl methacrylate resins are known to repel bacterial adhesion, suppress oral biofilms and acid production. Chemically stable and nonvolatile polymeric antibacterial agents exhibit antimicrobial activity by influencing and interrupting bacterial cell membranes [90].

#### 3.1.3. Nanoparticles in Orthodontic Elements

Some nanosized materials have antimicrobial properties [8,91,92]. This specification can be useful in orthodontic treatment in order to reduce caries and the number of microorganisms in the oral cavity. An increasing amount of bacterial strains become resistant to antibiotics, and bacteria have lower chances of developing resistance to metal nanoparticles [93] in comparison with conventional antibiotics. These information resulted in renewed interest in the use of alternative antibacterial agents such as metallic NPs: titanium dioxide (TiO_2_), gold (Au), silver (Ag), copper (Cu/CuO), silica (SiO_2_) and zinc oxide (ZnO) [8,94] or curcumin nanoparticles produced from underground stems of *Curcuma longa* [95]. Mirshashemi et al. [65] and Tahmasbi et al. [96] studied an effect of zinc oxide (ZnO) and chitosan (CS) nanoparticles used in the dental composite for orthodontic. Three different concentrations of this nanoparticle mixture—1, 5 and 10%—were considered in the experiment. The results showed that using composite contained 10% of nanoparticles led to a decrease in the formation of biofilm compared to unmodified composites.

In an in vivo research conducted by Metin-Gürsoy et al. [66], orthodontic brackets were coated with silver nanoparticles. It was observed that adding these nanoparticles was influential in *S. mutans* inhibition and decreased the caries in the smooth surface. Other nanoparticles with antimicrobial properties such as CuO are applicable in orthodontic adhesives to reduce microorganisms. According to a study carried out by Ghasemi et al. [10], it was observed that using silver and titanium oxide thin film on orthodontic stainless steel brackets can decrease the bacterial count (*S. mutans*); however, nano-titanium oxide film coated on the brackets increased the friction, which is unfavorable for orthodontic treatment.

Silver has been acknowledged for its antimicrobial properties against Gram-positive (including *Streptococcus mutans*)/negative bacteria, protozoa, some viruses, fungi and antibiotic-resistant strains [97,98,99].

To investigate the antiadherent and antibacterial properties of stainless steel (SS) and nickel titanium (NiTi) orthodontic archwires modified with silver against *Lactobacillus acidophilus*, Mhaske et al. [32] presented an example of a methodology that is widely used. The surface modification was carried out by thermal evaporation method. They performed two tests—the first one for the antiadherent property of surface-modified orthodontic archwires and the second one for their antibacterial property. Pure silver (99.9%) was used for obtaining a thin coating on archwires. To find out the results of the study, the weight of wires was compared to the initial one, as is shown in Table 2.

Antibacterial effects of the surface-modified archwires were carried out against lactobacilli. Firstly, the lactobacillus culture broth was diluted with MRS broth in order to obtain a proper optical density. Thereafter, the suspension was relocated in petri dishes containing uncoated and coated archwires. Antibacterial activity was characterized as an indicator of survival by colony-forming units (CFUs) for *Lactobacillus acidophilus*. The obtained results are shown in Table 3.

The results showed that silver coating applied to the orthodontic archwires prevented the *Lactobacillus acidophilus* adhesion and demonstrated antibacterial effects. Therefore, it can be concluded that silver coating can be used to protect the dental caries and dental plaque development during orthodontic treatment.

In 2012, Ryu et al. [65] developed a hard coating for stainless steel using silver-platinum alloys. The coating was created by physical vapor deposition. The antimicrobial properties of the Ag-coated samples or Ag release from the coated specimens were investigated by treating them to gram-positive *Streptococcus mutans* and *Aggregatibacter actinomycetemco-mitans*. After 16 h, the bacterial ingrowth level on the Ag-coated samples was decreased considerably (approximately 60%). The results confirmed that Ag-Pt coatings on load-bearing orthodontic bracket surfaces may ensure adequate antimicrobial activity and resistance to biofilm generation.

#### 3.1.4. Coating Orthodontic Archwires and Brackets with a Thin Film of Nitrogen-Doped TiO_2_ NPs

Photocatalysis of titanium dioxide (TiO_2_) on surfaces, compared with unmodified surfaces, tends to prevent the surface biofilms creation by improvements in hydrophilicity [94,100]. The nanosized TiO_2_ powder is difficult to disperse because of the intense accumulation caused by high surface energy. Therefore, it directly affects its antimicrobial and physiochemical properties [72].

In 2011, Shah et al. [40] assessed the antiadherent and antibacterial properties of surface-modified stainless steel of orthodontic brackets against *Lactobacillus acidophilus*. The surface modification was conducted by the radiofrequency magnetron sputtering method with photocatalytic TiO_2_. The bacterial strain was prepared in exactly the same way as in the above Mhaske’s experiment [32]. Uncoated SS brackets exhibited a higher weight increase (4.1%) compared to coated orthodontic brackets, in which a 2.6% weight change was noted.

To assess the antibacterial activity of the surface-modified brackets, the researchers calculated the survival rate by colony-forming in terms of CFUs for lactobacilli. Similar to the previous experiment, the bacterial strain was diluted with MRS broth in order to reach a proper optical density, and therefore was transferred onto to uncoated and coated brackets and illuminated with a UV-A black light inside the laminar air flow chamber. After that, the bacterial suspension was diluted and placed on MRS agar plates. The group containing TiO_2_ coating brackets has a statistically significant decrease in the survival rate of lactobacilli (CFUs) compared to uncoated stainless-steel brackets. In this study, researchers confirmed that the photocatalytic TiO_2_ demonstrated antiadherent properties and antibacterial effects against *Lactobacillus acidophilus* [40].

In 2014, Chhattani et al. [64] assessed the antiadherent and antibacterial properties of surface-modified stainless steel and nickel titanium orthodontic archwires against *Streptococcus mutans*. The surface modification was carried out by the sol-gel thin film dip coating method with photocatalytic TiO_2_. In this study, 80 specimens of each wires were used which were divided into eight groups (four groups in the antiadherent test and the other four in the antibacterial test). Groups including uncoated SS and uncoated NiTi wires were used as a control group for their according testing groups, containing coated SS and NiTi wires. The bacterial strain was prepared in exactly the same way as in the experiment conducted by Mhaske et al. [32] and Shah et al. [40]. Before the assessment of the bacterial adhesion, the wires were ultrasonicated to eliminate accidental macroscopic contamination and were dried in a desiccator. Thereafter, the wires were preweighted. An overnight-cultured *S. mutans* culture broth was inoculated in a sterile beaker containing 10 ml of a BHI broth to a final concentration of 10%. The methodology is similar to that in Mhaske’s research [32]. The weight comparison of the wires during the bacterial adhesion examinations is the following: uncoated SS wires showed a 35.4% increase in weight, whilst surface-modified wires only showed a 4.08% increase in weight. For NiTi wires, the increase in weight was 20.5% for uncoated wires and 4.4% for coated orthodontic archwires. Thus, uncoated wires exhibited a statistically significant weight increase in comparison with the surface-modified archwires. To assess the antibacterial properties of the surface-modified wires, the researchers calculated the survival rate by colony-forming in terms of CFUs for *S. mutans*. The following results were achieved: for SS wires—838.60 ± 48.97 (control group), 220.90 ± 30.73 (coated wires) and for NiTi wires—748.90 ± 35.64 (control group), 203.20 ± 41.94 (coated wires). The results showed that the groups consisting of coated wires exhibited a statistically significant decrease in the survival rate of *S. mutans* expressed as CFU compared with groups containing uncoated archwires.

In 2017, Liu et al. [15] showed the highest antibacterial activity for composite archwires (CAW) coated with N-doped TiO_2_ when compared to uncoated wires. In this study, Ti–44.73% NiTi SMA wire, Fe-18Cr-8Ni (SS) and pure Cu were used as the base metals. The NiTi SMA and SS wires were fixed end-to-end in a self-constructed apparatus with a pure Cu interlayer. A Nd:YAG laser welding system with a wavelength of 1064 nm was used for welding. TiO_2_ nanoparticle thin films were deposited by the RF magnetron sputtering method, with 99.99% ceramic TiO_2_ as the target. A mixture of Ar and N_2_ (gas flow ratio of Ar to N_2_ was 30:1) was used as the sputtering gas for N-doped TiO_2_ film and pure Ar was used as the sputtering gas for pristine TiO_2_ film. As a standard bacteria, *Streptococcus mutans* was chosen.

Shuai et al. [101] compared the antibacterial effects of thin films coated on stainless steel brackets. The study was carried out on four samples, with one of them as a control group exhibiting no antibacterial activity (uncoated samples). The group tested with N-doped TiO_2_ thin film showed the most effective antimicrobial ability, with a rate of 77.2%. The group filmed only with TiO_2_ showed a rate of 5.9% and the group coated with CAW indicated a rate of 0.9%, which means no bactericidal activity following the standard bacteria reduction criteria, which demonstrates that:

- Reduction under 20% results in no bactericidal activity;
- Reduction of 20–50% suggests low antibacterial activity;
- Reduction of 50–70% indicates strong bactericidal effect;
- Reduction above 70% is regarded as strong antibacterial activity.

In this study, the use of N-doped TiO_2_-coated archwires with strong bactericidal effect under visible light irradiation would kill the most *Streptococcus mutans* bacteria, which are the key factor responsible for caries. Therefore, the result shows that N-doped TiO_2_-modified compound arch wire has a high clinical practice potential.

**Figure 5:**
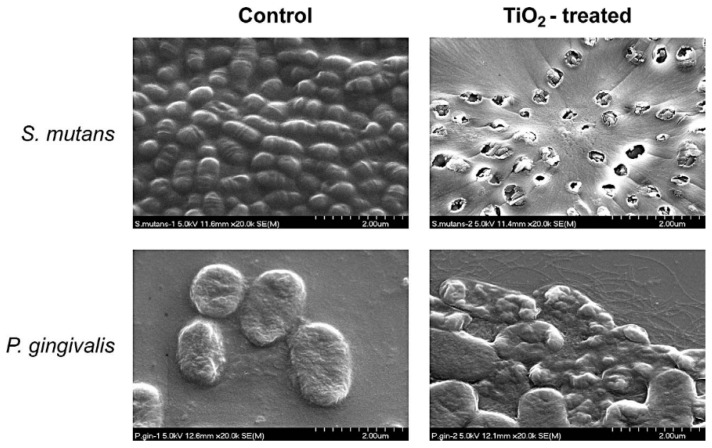
Scanning electron microscopy analysis of *Streptococcus mutans* and *Porphyromonas gingivalis* [11].

Chun et al. [11] assessed the antiadherent and antibacterial properties for the modification of orthodontic surfaces of stainless steel wires with photocatalytic titanium oxide (TiO_2_). Researchers conducted the experiment against two bacteria strains: *Streptococcus mutans* and *Porphyromonas gingivalis*. In this study, stainless steel wires were coated by TiO_2_ layer using the sol-gel dip-coating method in order to provide photocatalytic activity to the orthodontic wires. The antiadhesion properties of coated wires were evaluated by their weight change. The results showed that a 0.33% weight change was noticed in the TiO_2_-coated wires, whereas there was a 4.97% weight increase in the uncoated orthodontic wires. Apart the antiadherent ability of the photocatalyticTiO_2_-coated wires, antibacterial activities against *S. mutans* and *P. gingivalis* were indicated. In the dilution agar plate method, the survival rate of *S. mutans* was 100 CFU for the TiO_2_-coated wires, whereas it was 720 CFU in the control group (uncoated orthodontic wires). Since *P. gingivalis* is a strict anaerobe, the dilution agar plate method was not relevant for the antibacterial effect assay. Hence, the reduction in optical density at 660 nm was tested instead. In order to examine the antibacterial effect of TiO_2_-coated wires, the bacterial surface was observed by scanning electron microscopy. When the TiO_2_-coated wires were used with UV-A illumination, substantial damage was examined in nearly all *Streptococcus* cells. However, relatively low damage was observed in *P. gingivalis* (Figure 5).

Cao et al. [69] performed a study of assessing antimicrobial adhesive properties of a thin film of nitrogen-doped TiO_2_ NPs. Stainless steel orthodontic brackets were used as a basic material. The nitrogen doping and modification allow TiO_2_ to demonstrate catalytic activity in the visible-light area. In this experiment, good antiadhesive properties against *Streptococcus mutans* were observed. The antimicrobial activity levels of the coated bracket against *Streptococcus mutans*, *Lactobacillus acidophilus*, *Actinomyces viscous* and *Candida albicans* were 95%, 91%, 69% and 99%, accordingly. These findings indicate that this coating can be useful in the protection against enamel demineralization and gingivitis during orthodontic therapy.

**Figure 6:**
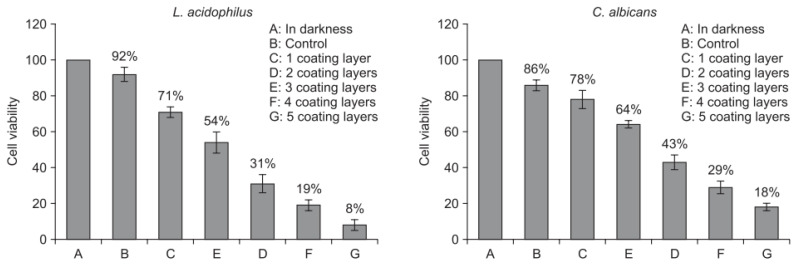
Antibacterial effect of TiO_2_ thin film with 1–5 coating layers against *Lactobacillus acidophilus* and *Candida albicans* [69].

Cao et al. [69] conducted the experiment on cer0amic orthodontic brackets coated with TiO_2_ thin films to assess antimicrobial properties. The sol-gel dip coating technique was used to create TiO_2_ thin films on ceramic brackets. The antibacterial activities of particular thin films were measured against *Lactobacillus acidophilus* and *Candida albicans* by a colony counter. In this study, researchers also checked dependence between the number of layers and the microstructure of thin films calcined and the antibacterial activity (Figure 6). It was proved that the layer number did not affect the microstructure of thin films calcined but decreased the bacteria cell viability.

There is a noticeable reduction in cell viability at 8% for Lacidophilus and 15% for C. albicans using a thin film with five coating layers, which showed the strongest antimicrobial activity on each microbes in comparison to the other samples.

### 3.2. Mechanical Properties of Coating for Orthodontics Elements

This section has been divided into three subsections depending on the properties and characteristics of all types of coatings used for orthodontic archwires [17,27]: (1) Mechanical properties of load and deflection (the three-point bending test); (2) Properties of the surface roughness and the mechanical sliding mechanism; (3) Corrosion resistance.

#### 3.2.1. Mechanical Properties of Load and Deflection (the Three-Point Bending Test)

The orthodontic archwires fabrication depends on their material composition and the geometry of its cross-section. Certain variations in its cross-section might influence on the mechanical properties of the orthodontic wire in terms of torsion and rigidity and on its final clinical performance [102]. Archwire with a round cross-section generates low forces, so that low friction and wear have occurred in the lock slot. Wires with a rectangular cross-section induce high forces and—due to their complete adjustment to the gap of the brace—provide full control of tooth movement [103].

**Figure 7:**
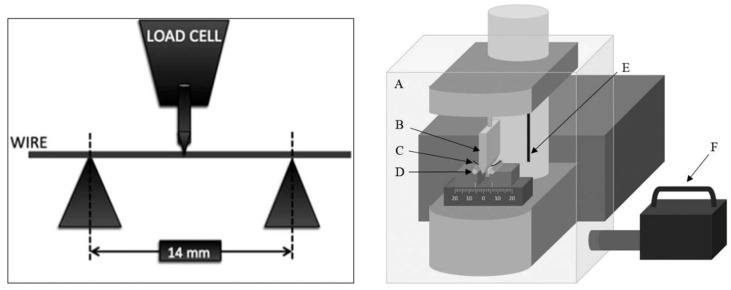
Three-point bending test—scheme; A: thermal screen, B: indenter, C: wire sample, D: supporting points, E: temperature sensor, F: hot-air circulation temperature controller [106,107].

The mechanical properties might be examined by the three-point bending test, for which a jig with two parallel brass rods can be constructed. Two central incisor brackets are bonded on the top of these rods with an inter bracket distance of 14 mm (Figure 7). This test evaluates the load-deflection property that is very important for the nature of tooth movement and gives information about the behavior of the wires. The advantage of this test is the ability to get mechanical simulation similar to clinical application comparing different orthodontic archwires to super-elastic properties [104]. Through three-point test bending, it is possible to analyze several parameters, such as the load curve, the discharge curve, the modulus of elasticity, the form of resiliency or the material’s ability to store energy when it is deformed [22,105]. The three-point bend tests produced load-deflection diagrams consisting of an upper loading curve and lower unloading curve. There is a difference between these curves, which represents hysteresis in the material.

Da Silva et al. [25] analyzed four groups of orthodontic wires: uncoated archwires, which acted as a control group; coated orthodontic wires with a cross-sectional dimensión; wires with all the surfaces completely coated; archwires of which only the vestibular surface had been coated. Five features were evaluated: inner wire dimensions, modulus of elasticity, modulus of resilience, maximum deflection force and load deflection curve characteristics. Each samples were examined in a universal testing machine in a three-point bending test. The results showed that wires producing smaller forces of loading and unloading, possessed a higher modulus of elasticity, a lower modulus of resilience and also demonstrated a minimum values of bending than the control group.

In another study, it was confirmed that ultraesthetic coated archwires (GandH, Greenwood, Ind) created smaller loading and unloading forces than uncoated wires with identical nominal dimensions. With the Damon2 passive self-ligating brackets (Ormco, Orange, Calif, USA), retrieved rounded archwires exhibited no considerable difference in loading and unloading forces compared to the as-received esthetic archwires at different loading and unloading forces. The authors stated that this situation appeared to result from the fact that passive self-ligating braces caused low friction and were not affected by the high surface roughness and detoriation of the coating [33].

In the study performed by Matias et al. [108], the load-deflection properties of coated nickel-titanium (NiTi) and esthetic archwires combined with conventional ceramic brackets were compared using a clinical simulation device. In this experiment, the following materials were used: non-coated NiTi (NC), Teflon-coated NiTi (TC), rhodium-coated NiTi (RC), epoxy-coated NiTi (EC), fiber- reinforced polymer (FRP), and three different conventional brackets: metal-insert polycrystalline ceramic (MI-PC), polycrystalline ceramic (PC) and monocrystalline ceramic (MC). The results showed the decreasing force ranking of archwires: rhodium-coated NiTi (RC), non-coated NiTi (NC), Teflon-coated NiTi (TC), epoxy-coated NiTi (EC) and fiber-reinforced polymer (FRP). During unloading at 3 mm, the FRP wire was plastically deformed and generated an extremely low force in 2; 1 and 0.5 mm of unloading.

In 2015, Washington et al. [28] carried out research on the differences in loading and unloading forces provided by six coated nickel-titanium wires and their noncoated counterparts. The wires were examined using a three-point bending test. No statistically important changes in force rate between coated and uncoated wires were observed.

In the study performed by Bradley et al. [26], three-point bending tests compared the mechanical properties of two types of aesthetic coated nickel-titanium (NiTi) wires with comparable regular NiTi wires in the as-received state and after clinical use. The results showed statistically significant differences in all activation and deactivation stiffnesses and forces between a noncoated and coated wire group. There were no differences in bending values between the uncoated NiTi wires; however, the coated types exhibit statistically relevant differences in the as-received state and after clinical use in a majority of stiffness and force parameters.

#### 3.2.2. Properties of the Surface Roughness and the Mechanical Sliding Mechanism

**Figure 8:**
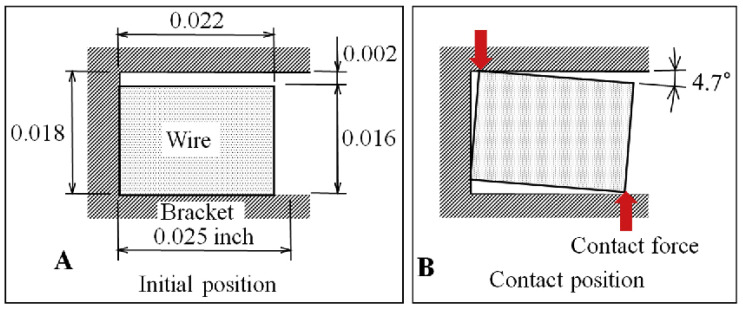
The archwire position in the brackets slot: an initial position (**A**), maximum wire displacement and contact points—greatest abrasive wear areas (**B**) [111].

Another important issue for orthodontic elements is surface roughness. The surface topography of an orthodontic archwire is an essential property, which is known to influence its mechanical characteristics, corrosion behavior, esthetic appearance and its biocompatibility. Clinically, a rough surface promotes a larger plaque accumulation what affects its friction properties, and increases corrosion and color instability [109]. Contact between two elements in a bracket slot (Figure 8), during orthodontic treatment, only increases this friction process and wear [75,110]. What is more, a still-changing oral environment, rich in typical microflora and the composition of consumed food products and observed cooperation between wire and bracket, induce a complex process of material erosion, the so-called bio-tribocrosion process [110].

Many methods are used to assess the surface roughness, such as atomic force microscopy, spectroscopy or contact surface profilometry.

The physical and mechanical properties such as sliding ability of the wire are affected by negative impacts of corrosion phenomena [4]. There are many different materials and methods used to improve the surface of the archwire and orthodontic brackets. Recent studies have shown that the orthodontic elements with coatings are also characterized by lower resistance to sliding. In 2012, Farronato et al. [112] conducted a study which evaluated in vitro the effect of Teflon coating on the resistance to sliding of orthodontic archwires. This was carried out by using frictional resistance tests by means of a universal testing machine and compared with conventional uncoated wires. The results indicated that Teflon coating has the capability to decrease the sliding resistance of orthodontic archwires, because, for all bracket–archwire combinations, Teflon-coated archwires showed reduced friction compared to the equivalent uncoated wires.

In 2013, da Silva et al. [25] assessed the surface characteristics and coating stability of esthetic-coated archwires after 21 days exposure to the oral environment. Thereafter, they were compared to those of conventional stainless steel and nickel titanium ones. Surface measurements were made by an atomic force microscope with a noncontact tip coated with silicon. The results showed that the remaining coating had significantly deteriorated and an increased surface roughness compared to their post-clinical-control counterparts (conventional SS and NiTi wires).

Zhang et al. [113] carried out a study to compare the effects of nanostructured diamond-like carbon (DLC) coating and nitrocarburizing on the frictional properties of orthodontic stainless steel wires. The DLC thin film was applied on the surface of stainless-steel wire by the chemical vapor deposition method. The frictional force created by each archwire–bracket combination was examined under dry conditions using a frictional testing apparatus mounted on the crosshead of a universal testing machine. The results showed that a nanostructured DLC coating and nitrocarburizing processes both considerably reinforced the surface hardness and minimized the friction of stainless-steel wires and provided improved corrosion resistance and greater elasticity.

In 2017 Muguruma et al. [107] analyzed the coatings covering esthetic orthodontic wires and the influence of such coatings on bending and frictional properties. In this study, four commercially available coated esthetic wires were used. To evaluate the external surfaces of archwires, atomic force microscopy was used. The static frictional force triggered with every configuration of wire-bracket was carried out under dry conditions, using a custom-fabricated friction-testing device connected with the universal testing machine. The researches confirmed that the friction of the coated wires was influenced by the total cross-sectional and inner core dimensions, inner core elastic modulus, inner core nano-hardness, and elastic modulus, but not by surface roughness.

Redlich et al. [114] reduced friction between coated orthodontic stainless steel archwires and coated brackets during tooth movement by coating the wire with nickel-phosphorous electroless film impregnated with inorganic fullerene-like nanoparticles of tungsten disulfide (IF-WS2). The analysis of coated wires was carried out with scanning electron microscope and energy-dispersive X-ray spectrometer. Friction tests simulating archwire functioning of the coated and uncoated wires were conducted by an Instron machine. The results showed that the friction forces on the coated wire were decreased by up to 54%. It was proven that the wires coated with these nanoparticles might offer a new possibility to significantly decrease friction during tooth movement.

However, studies carried out in vivo showed that the clinical use of coated orthodontic archwires increases their surface roughness and the friction level [22,115]. The latest technological development in the orthodontic research regards the high aesthetic value of orthodontic appliances. This led to the production of a polymer wire with a high ductility and considerable elastic return [116]. Bradley et al. [26] developed a new thermoplastic polymer for orthodontic appliances. They assessed a three-point bending test, tensile, friction, stress relaxation behavior and formability characteristics. The results showed that the stresses delivered were generally lower than typical nickel-titanium wires and the friction coefficients were lower than metallic conventional wires, which improved the slipping mechanism with brackets.

#### 3.2.3. Corrosion Resistance

Orthodontic appliances are exposed to corrosion in the oral environment, which is an ongoing process of degradation induced by wide fluctuations of factors such as pH, temperature, microbial, and salivary factors. The corrosion of nickel titanium (NiTi) archwires causes serious misgivings due to their high nickel concentration (47% to 50% nickel) and related biocompatibility challenges. Since nickel atoms are not strongly held in the NiTi alloy as in other intermetallic compounds, its release into the oral cavity during clinical use is reported to cause several types of adverse reactions ranging from mild hypersensitive responses to extremes of cytotoxic, mutagenic, and carcinogenic changes. NiTi arch wires are constantly exposed to the oral cavity and a carry greater risk of toxic effects. Corrosion also affects the physical properties of NiTi wires, including their crucial shape memory and superelasticity features. Moreover, corrosion products can increase wire-bracket frictional coefficients and decelerate tooth movement. In acidic pH, hydrogen ions penetrate into the NiTi alloy and form brittle titanium hydrides, which cause wire fracture. Similarly, fluoride ions present in various anti-caries preparations are also known to increase their susceptibility to corrosion [79,117,118].

The location of orthodontic wires in the oral cavity environment for a long time is associated with their corrosivity and ion release. In this environment they should be corrosion-resistant and prevent the ion release, but they should not generate allergic reactions. This means that orthodontic archwires have to be biocompatible with oral cavity [4]. The most commonly used method to evaluate the corrosion resistance is the electrochemical testing used for the potentiostatic anodic polarization experiment [118].

Krishnan et al. [118] compared the corrosion performance of five types of surface modified nickel titanium (NiTi) wires, available on the market, with a conventional NiTi by degradation potential in an anodic polarization scan in Ringer’s solution. Surface features were examined by scanning electron microscopy, atomic force microscopy, and energy-dispersive analysis. Surface modification of NiTi wires showed effectiveness in decreasing the surface roughness and improving their resistance to corrosion.

**Figure 9:**
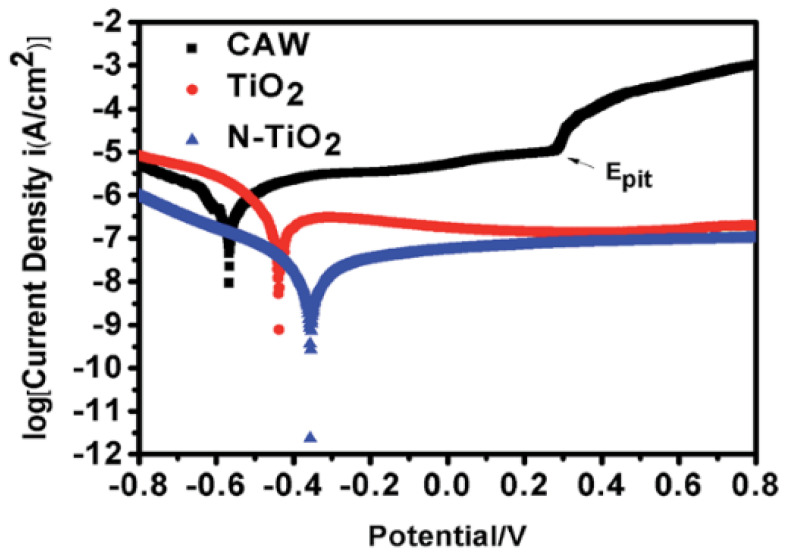
Potentiodynamic polarization curves of CAW, TiO_2_ coat-CAW and N-TiO_2_ coated CAW [15].

In 2017, Liu et al. [15] found that corrosion resistance of composite archwire (CAW) might be substantially upgraded by coating with the TiO_2_ nanocrystal thin film, especially using the N-doped TiO_2_ nanocrystal thin film, which is crucial for its clinical application. Figure 9 shows three typical polarization curves of unmodified CAW, TiO_2_-coated CAW and N-TiO_2_-coated CAW in artificial salvia solutions. The results clearly showed that the TiO_2_- and N-TiO_2_-coated CAWs may significantly enhance the corrosion resistance of pristine CAW in artificial saliva. It was showed that N-doped TiO_2_ film exhibited a significantly higher anticorrosion potential than the TiO_2_ film.

The corrosion of the orthodontic wires’ coating after clinical use in the oral environmental might also lead to undesirable results, for example, microstructural and chemical changes which can cause a mechanical deformation of the archwire and the release of chemical ions within the oral fluids [100].

## 4. Discussion

Recent progress in the area of orthodontic coatings enabled the development of a wide range of coatings, which possess an incredible spectrum of properties. Although the use of nanoparticles, such as TiO_2_ or nano-silver, in orthodontics, may bring new opportunities, the performed studies carried out the antimicrobial, mechanical or physical properties in a relatively short period of time, i.e., from 24 h to a week. In addition, it should be considered the constraints of tests conducted mainly in vitro. There are no data on the long-term efficacy of orthodontic material based on this technology and further testing is required, as a safety issues which may be related to nanoparticle size. Since a part of the nanoparticles could be released from the coating by friction processes during sliding mechanism between archwire and brackets, it was essential to investigate the coated wires after sliding to ensure the adhesion properties of the nanocremics in the basic layer. The presence of the nanoparticle is a necessary condition for correct lubrication during movement. This makes perspectives for the future research and innovations.

The surface roughness is suggested not to be a decisive criterion for assessing the corrosion resistance for surface-modified NiTi archwires. The type and nature of coating materials such as nitride ions, metals, oxides, Teflon or resins had exhibited a stronger impact on determining the potential for corrosion of NiTi wires compared to the values of surface roughness.

The technology of applying layers on the orthodontics wires, such as sol-gel method or radiofrequency magnetron sputtering are widely used in covering surfaces of various shapes with thin layers. By using the appropriate compositions, the coatings can be modified to give them the appropriate porosity or hardness or to adjust their mechanical, physical or chemical properties or antibacterial protective.

## 5. Conclusions

Adequate implementation of each type of coated archwire can improve patients’ comfort as well as reducing the treatment time. The personal orthodontist should always know and understand the needs and opportunities during each stage of treatment. It needs to use the desired features of a specific coated archwire type which has been chosen to satisfy the requirements of the current clinical situation. This would allow for the most optimal and efficient treatment results.

Coating the orthodontic archwires using the discussed methods, as well as modifying their surface with additives, improves their mechanical properties and can minimize bacterial adhesion on the surface. Appropriate coating techniques enable the reduction in corrosivity, wear and tear, as well as its delamination. The material used to the coating may accelerate orthodontic treatment. The analysis of additives such as TiO_2_, ZnO, Ag and silver nanoparticles as well as uncoated arches can compare antimicrobial activity and environmental resistance. The layer thickness of coatings applied to the archwires is also relevant for the antibacterial effect.

Further developments regarding the coating methods and compositions, which permits their use in the oral cavity, could reduce the friction problem during orthodontic treatment and release nickel ions, minimizing the treatment time and the risk of bacteria adhesion.

## Figures and Tables

**Table 1:** Summary on studies on the antiadherent and antibacterial properties of orthodontic materials and coatings for orthodontic elements.

Material /Substrate	Coating Composition	Coating Technique	Bacteria/Microbial Strains	Methods	Results	Ref.
stainless steel (SS) and NiTi archwires	pure silver (99.9%)	Thermal evaporation method	*Lactobacillus acidophilus* (LA)	Antiadherent was evaluated by the weight change in the wires; Antibacterial by the dilution agar plate method.	The silver coating prevented the adhesion of *L. acidophilus* and demonstrated antibacterial effect against bacteria.	Mhaske et al. [32]
SS brackets	photocatalytic TiO_2_	Radiofrequency magnetron sputtering	*Lactobacillus acidophilus*	Antiadherent was evaluated by the weight change in the brackets and antibacterial the dilution agar plate method.	The photocatalytic TiO_2_ coating prevented the adhesion of *L. acidophilus* and demonstrated antibacterial effect against bacteria.	Shah et al. [40]
SS and NiTi archwires	photocatalytic TiO_2_	Sol-gel thin film dip-coating	*Streptococcus mutans* (SM)	Antiadherent was evaluated by the weight change in the wires; Antibacterial by the dilution agar plate method.	The photocatalytic TiO_2_ coating prevented the adhesion of *S. mutans* and demonstrated antibacterial effect against bacteria.	Chhattani et al. [64]
composite archwires (CAWs): NiTi and SS wires	99.99% ceramic TiO_2_ and N-doped TiO_2_	Radiofrequency magnetron sputtering	*Streptococcus mutans*	Antiadherent was evaluated by the weight change in the wires; Antibacterial by the dilution agar plate method.	Wires coated with N-doped TiO_2_ thin film showed the most effective antimicrobial effects.	Liu et al. [15]
SS orthodontic wires	photocatalytic TiO_2_	Sol-gel thin film dip-coating	*Streptococcus mutans* and *Porphyromonas gingivalis*	Antiadherent was evaluated by the weight change in the wires; Antibacterial by the dilution agar plate method for *Streptococcus mutans* and by spectrophotometry for *Porphyromonas gingivalis.*	The TiO_2_-coated orthodontic wires showed an antiadherent effect against *S. mutans* and bactericidal effect on *S. mutans* and *P. gingivalis.*	Chun et al. [11]
orthodontic composite	Chitosan (CS) and zinc oxide (ZnO)nanoparticles (NPs)		*Streptococcusmutans, S. anguis* and *Lactobacillus acidophilus*	Antibacterial by the dilution agar plate method.	Antibacterial by the dilution agar plate method.	Mirhashemi et al. [65]
orthodontic brackets	Nanosilver	beam evaporation method	*Streptococcus mutans*	Antibacterial was evaluated by the dilution agar plate method.	Nanosilver coated orthodontic bracket favoured the inhibition of *S. mutans* on day 30 and reduction of caries on the smooth surfaces.	Metin-Gürsoy et al. [66]
SS surfaces	Ag and Ag-Pt alloys	physical vapor deposition	*Streptococcus mutans* and *Aggregatibacter actinomycetemo-mitans*	Antibacterial was evaluated by the dilution agar plate method.	The coatings released sufficient Ag ions when immersed in phosphate-buffered saline and showed antimicrobial effect on *S. mutans* and *Aggregatibacter actinomycetemcomitans* strains.	Ryu et al. [67]
SS brackets	TiO_2_	physical vapor deposition	correlation of coating adhesion and surface roughness	The adhesiveness of TiO_2_ coating was assessed quantitatively and qualitatively by measuring hardness by micro Vickers.	Surface roughness correlates with coating adhesion, if the surface roughness increased then the adhesion of coating would be decreasing.	Supriadi et al. [68]
ceramic brackets	photocatalytic TiO_2_	sol-gel dip coating	*Lactobacillus acidophilus* and *Candida albicans*	The antibacterial effect was measured by the numbers of colonies these bacteria strains on a plate and by the photocatalytic antibacterial test under UV-A light irradiation.	The films with 5 coating layers and annealed at 700 °C exhibited the greatest antibacterial activity against *L. acidophilus* and *C. albicans* under UV-A light irradiation.	Cao et al. [69]
NiTi and Cu-NiTi archwires	without coating		*Streptococcus mutans*	The *S. mutans* adhesion was evaluated by using real-time polymerase chain reaction.	*S. mutans* adhesion, surface roughness, and surface free energy were greater in Cu-NiTi than NiTi archwires.	Abraham et al. [70]
NiTi archwires after 4 and 8 weeks of intraoral use	- Esthetic coated NiTi wires: - Ortho Organizers (Sao Marcos, Calif) - Forestadent (Pforzheim, Germany) - TP Orthodontics (Laporte, Ind)		*Streptococcus mutans, Staphylococcus aureus* and *Candida albicans*	The amount of bacterial adhesion was quantified using the colony-count method.	Biofilm adhesion increased after intraoral use at all time intervals. There was a positive correlation between surface roughness and biofilm adhesion in vivo only.	Taha et al. [71]
SS brackets	nitrogen-doped (N-doped)	Radiofrequency magnetron sputtering	*Streptococcus mutans*, *Lactobacillus acidophilus*, *Actinomyces viscous* and *Candida albicans*	The antimicrobial activity was assessed on the basis of colony counts.	The bracket coated with thin film shows high antimicrobial activity against bacteria and strongly prevents the adherence of them.	Cao et al. [72]
Ti and TiAg metals	photocatalytic TiO_2_	anodic oxidation (AO) and thermal oxidation (TO)	*Streptococcus mutans*	Photocatalytic antibacterial activity with ultraviolet A (UVA) illumination.	The antibacterial effect of a TiO_2_ film formed by AO superior to that formed by TO. The addition of Ag to Ti specimen indicated a synergistic effect on the photocatalytic antibacterial property against *S. mutans.*	Choi et al. [73]
SS, ceramic and plastic orthodontic brackets	without coating		*Streptococcus mutans* and *Streptococcus sanguis*	Adhesion was quantitated by a microbial culture technique by treating the brackets with adhering bacteria with trypsin and enumerating the total viable counts of bacteria recovered after cultivation.	There were no differences in the adherence to stainless steel, ceramic, or plastic brackets. The presence of an early salivary pellicle and *S. sanguis* reduced the number of adhering *S. mutans* to all three types of brackets.	Papaioannou et al. [6]
4 different kinds of esthetic wires *Ultraesthetic, Dany Coated, Bioforce Sentalloy White, TruGold* and 2 uncoated: SS and NiTi wires	- Epoxy-coated nickel-titanium alloy - Bio-polymer (outer) and silver (inner) - coated nickel-titanium alloy - Rhodium-coated nickel-titanium alloy - 24K gold-plated stainless-steel		*Streptococcus mutans* and *Streptococcus sanguis*	The amount of bacteria adhering to the wires was quantified using the colony-counting method and by observing by scanning electron microscopy.	Adhesion of *S. mutans* to wires was greater than that of *S. sobrinus*. Some esthetic coatings on NiTi alloy might reduce *S. mutans* SM adhesion in vitro in the short term.	Kim et al. [23]
a conventional orthodontic composite material (Transbond XT) + experimental composite adhesive material (ECA) and two conventional adhesives (composite and resin-modified glass ionomer)	ECA was modified by the addition of silica nanofillers and silver nanoparticles		cariogenic *Streptococci*	Effect of surface characteristics, physical properties, and antibacterial activities of ECA against cariogenic *Streptococci*.	ECA had rougher surfaces than conventional adhesives due to addition of silver nanoparticles and bacterial adhesion to ECA was less than to conventional adhesives. No significant difference in shear bond strength and bond failure interface between ECA and conventional adhesives were noted.	Uysal et al. [74], Ahn et al. [7]

**Table 2:** Groups of archwires used for the antiadherent properties of surface-modified orthodontic archwires and comparison of weight: initial, final and changes in weight of wires [32].

Group		Weight			Significance
		Initial	Final	Change	
1. Control group	Includes 10 uncoated stainless steel orthodontic wires	0.240 ± 0.021 (0.200–0.250)	0.325 ± 0.035 (0.250–0.350)	0.085 ± 0.024 (35.4%)	*p* < 0.001 **
2. Experimental group	Includes 10 surface-modified stainless steel orthodontic wires coated with silver	0.245 ± 0.028 (0.200–0.300)	0.255 ± 0.037 (0.200–0.300)	0.010 ± 0.020 (4.08%)	*p* = 0.168
3. Control group	Includes 10 uncoated nickel titanium wires	0.220 ± 0.026 (0.200–0.250)	0.265 ± 0.033 (0.250–0.350)	0.045 ± 0.028 (20.5%)	*p* < 0.001 **
4. Experimental group	Includes 10 surface-modified nickel titanium orthodontic wires coated with silver	0.225 ± 0.042 (0.150–0.300)	0.235 ± 0.047 (0.150–0.300)	0.010 ± 0.021 (4.4%)	*p* = 0.169

** Statistically significant.

**Table 3:** Groups of archwires used for antibacterial property of surface modified orthodontic archwires and comparison of colony count [32].

Group		Colony Count Range	Mean ± SD	*p* Value
1. Control group	Includes 10 uncoated stainless steel orthodontic wires	776–934	836.60 ± 48.97	*p* < 0.001 **
2. Experimental group	Includes 10 surface-modified stainless steel orthodontic wires coated with silver	176–262	220.90 ± 30.73	-
3. Control group	Includes 10uncoated nickel titanium wires	710–810	748.90 ± 35.64	*p* < 0.001 **
4. Experimental group	Includes 10 surface-modified nickel titanium orthodontic wires coated with silver	117–264	203.20 ± 41.94	-

** Statistically significant.
